# Evidence for Dietary Management of Histamine Intolerance

**DOI:** 10.3390/ijms26189198

**Published:** 2025-09-20

**Authors:** Kirsten Jackson, Wendy Busse, Patricia Gálvez-Martín, Andrea Terradillos, Daniel Martínez-Puig

**Affiliations:** 1 The IBS Dietitian; kirsten@theibsdietitian.com; 2 Land and Food Systems, University of British Columbia, Vancouver, BC V6T 1Z4, Canada; wendy.busse@ubc.ca; 3 R&D Bioiberica S.A.U., E-08389 Palafolls, Spain; aterradillos@bioiberica.com (A.T.); dmartinez@bioiberica.com (D.M.-P.)

**Keywords:** histamine, histamine intolerance, food intolerance, diamine oxidase (DAO)

## Abstract

Self-reported food intolerances are estimated to affect between 15–20% of the population. Among them, histamine intolerance (HIT) has emerged as a focus of particular interest. It is defined as a disequilibrium between dietary histamine and the capacity of the organism to degrade intestinal histamine, leading to the appearance of intestinal and extra-intestinal symptoms. HIT is thought to be associated with low activity or blockade of diamine oxidase (DAO), the main enzyme for histamine degradation. The diagnosis is hampered by the lack of a validated biomarker and is mainly based on clinical assessment and response to a low histamine diet and reintroduction. The therapeutic approach is centered on dietary management, restricting foods that may increase circulating histamine levels. DAO supplementation has been shown to potentially contribute to histamine degradation in the intestinal lumen, but its activity varies depending on the presence of cofactors and the enzyme’s origin. Limited clinical evidence reflects the difficulty of dietary management and suggests a beneficial role of DAO supplementation on the clinical manifestations associated with HIT.

## 1. Introduction

Food intolerance is a common condition as it is estimated to affect 15–20% of the population [1]. Unlike food allergies, food intolerance mechanisms are not immune-mediated and could derive from chemicals with potential pharmacological activity, enzyme deficiencies or non-specific gut and non-gut reactions to food including irritable bowel syndrome and other functional disorders of gut–brain interaction [2,3,4].

Prevalence estimates are hampered by the coexistence of diverse diagnostic approaches, aimed to differentiate conditions with different mechanisms and clinical presentations, in which the most common symptoms are gastrointestinal [5].

**Figure 1:**
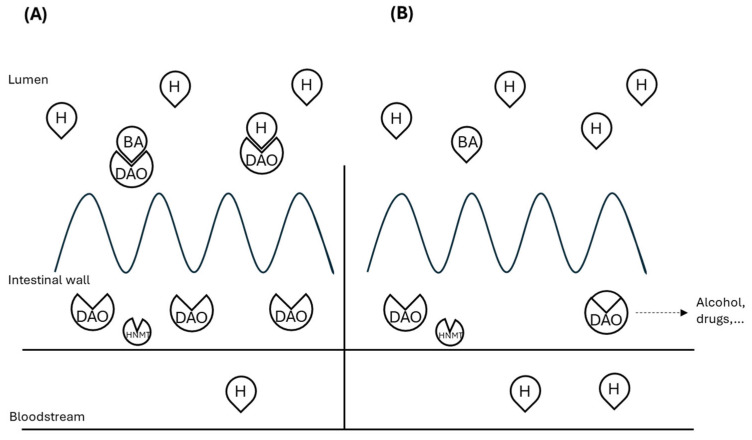
Scheme of histamine degradation in the intestinal mucosa of (**A**) healthy individuals or (**B**) histamine intolerance. H: histamine; BA: biogenic amines; DAO: diamine oxidase; HNMT: histamine-N-methyl transferase. Adapted from [20,21].

Among food intolerances, histamine intolerance (HIT) has emerged as a focus of particular interest during the last years [6]. It is defined as a disequilibrium between dietary histamine and the capacity for histamine degradation in the gastrointestinal tract [7,8]. Diamine oxidase (DAO), which is synthesized by mature enterocytes located in the upper intestinal villi [9], is the main enzyme for histamine degradation [10,11]. Its high expression in the gastrointestinal tract might protect from histamine present in contaminated food or generated by bacteria within the gut microbiome [12] (Figure 1). It is assumed that HIT symptoms are a direct consequence of reduced intestinal DAO activity, similar to lactose intolerance [8]. Reduced DAO activity may result in excess histamine reaching systemic circulation and triggering a variety of non-specific intestinal and extra-intestinal symptoms such as bloating, diarrhea, headache, dizziness, urticaria or rhinorrhea among others [13]. DAO deficiency can have a genetic background, as certain polymorphisms for genes encoding the DAO enzyme have been associated with HIT symptoms [14,15]. Specifically, the most relevant single-nucleotide polymorphisms (SNPs) of the AOC1 gene leading to reduced DAO enzyme activity in the Caucasian population are c.47C>T (rs10156191), c.995C>T (rs1049742), and c.1990C>G (rs10449793) [16,17]. In addition, gastrointestinal disorders compromising the integrity of the intestinal mucosa may lead to DAO deficiency [18]. In this regard, data have been published linking the imbalance or dysbiosis of the intestinal microbiota in patients with HIT with an overabundance of histamine-secreting bacteria (*Enterococcus faecalis, Bifidobacterium pseudocatenulatum, Lactobacillus gasseri, Escherichia coli, Morganella morganii* and *Proteus mirabillis*) [19]. Thus, intestinal dysbiosis in patients diagnosed with HIT could contribute to mucosal inflammation, potentially altering DAO activity.

In addition, HIT has been described concomitant to other conditions like lactose or fructose malabsorption [22]. It has been suggested these conditions could be also associated with damage of the small intestinal mucosa. Besides this, the activity of DAO is affected by widely used drugs such as clavulanic acid, chloroquine, or cimetidine [23], or other substances like alcohol or biogenic amines [7].

There is limited information regarding gender differences in DAO deficiency. In a recent study, no differences were found in the prevalence of DAO deficiency determined by single-nucleotide polymorphism (SNP) variants of the AOC1 gene between male and female newborns [24]. Studies evaluating serum DAO levels have obtained mixed results. While some studies have reported no gender differences [25,26], one study reported higher DAO concentrations in men as compared to women [16]. Serum DAO levels are affected by the menstrual cycle in premenopausal women, being higher in the luteal phase as compared with the follicular phase [27]. An estrogenic regulation of DAO activity has been reported in different animal models [28,29]. In addition, estrogen increases mast cell reactivity [30] and enhances histamine dependent skin-prick test reactions [31]. In this regard, HIT symptoms could potentially be affected by the menstrual cycle or menopause. Data on age differences in DAO deficiency is limited. Typically, clinical symptoms of HIT are more often presented in middle-aged people [20]. In fact, it has been estimated that 80% of the patients are adults [7], but there is also evidence of DAO deficiency in pediatric patients with gastrointestinal symptoms [32]. In contrast to the adult population, the limited available evidence suggests gender differences in the pediatric population, the prevalence being higher in males [33].

Dietary management is considered the cornerstone for the treatment of HIT. Dietary recommendations are oriented towards a reduction of histamine-rich foods; foods rich in other biogenic amines, mainly putrescine and cadaverine; and in some cases, foods that have been suggested to have histamine-releasing capacities [7,21], although their potential mechanism of action has not been described and there are discrepancies about the scientific evidence to support their exclusion [34,35]. Consequently, in clinical practice, there is confusion due to discrepancies on the histamine content of some foods [36] as well as on the potential histamine-releasing capacity of others [34].

Besides low-histamine diets, oral supplementation with exogenous DAO enzyme has been proposed to contribute to overall dietary histamine degradation at the intestinal level [8,21]. Although efficacy has been demonstrated in some intervention studies [37,38,39], some aspects of oral DAO supplementation have not been fully elucidated, such as the absence of intestinal absorption, kinetics, and comparative efficacy, depending on the enzyme’s origin.

The aim of the present review is to propose a diagnostic approach for HIT, review the therapeutic approaches available, and provide practical recommendations for its dietary management.

## 2. Molecular Aspect

**Figure 2:**
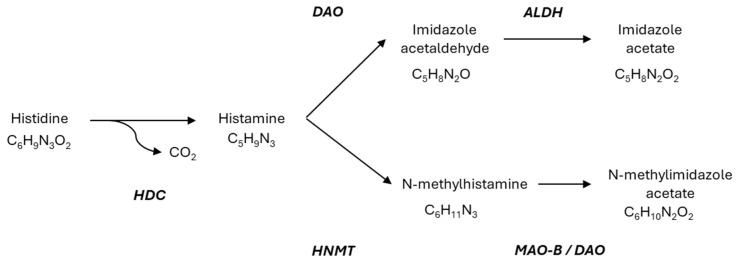
Summary of histamine synthesis and metabolism. HDC: histidine decarboxylase; DAO: diamine oxidase; HNMT: histamine-N-methyl transferase; ALDH: aldehyde dehydrogenase; MAO: monoamine oxidase. Adapted from [6,7,21].

Histamine (2-[4-imidazolyl] ethylamine) is a bioactive amine produced through the decarboxylation of the amino acid L-histidine, a reaction catalyzed by the enzyme L-histidine decarboxylase (Figure 2) [21].

Histamine is widely distributed throughout the body, mainly in the lungs, skin, and gastrointestinal tract. Histamine is predominantly produced by mast cells and basophils. It is also found in significant amounts in gastric enterochromaffin-like cells, as well as in platelets, histaminergic neurons, and lymphoid tissues such as lymph nodes and the thymus. In these cells, L-histidine undergoes decarboxylation via the enzyme histidine decarboxylase (HDC) within the Golgi apparatus, resulting in the formation of histamine. Once synthesized, histamine is sequestered in cytoplasmic granules. Upon cellular sensitization and subsequent degranulation, histamine is released into the surrounding environment [40].

Histamine acts as a key mediator in multiple physiological and pathological processes, including allergic reactions, inflammation, neurotransmission, and immune modulation. Its effects are primarily exerted through the activation of four types of receptors: H1R, H2R, H3R, and H4R [41,42]. Among them, H1R is broadly expressed across various tissues, including epithelial cells, smooth vascular muscle, neurons, glial cells, and cells of the immune system. Upon activation by histamine typically released from mast cells or basophils, H1R mediates key physiological responses associated with inflammation and allergic reactions. Due to its central role in these processes, H1R has been a major pharmacological target in the development of antihistamine therapies [6,43].

Regulation of H1R signaling occurs at both the desensitization and transcriptional activation levels. Downregulation occurs through receptor phosphorylation-dependent desensitization [44,45]. Regarding other histamine receptors, H2R is present in different cells including stomach parietal cells, enterocytes, vascular smooth muscle cells and lymphocytes [46]. H3R is predominantly localized in the brain [47] and H4R is mainly expressed in hematopoietic cells [48]. H2R is linked to the activation of adenylate cyclase, while H3R functions through Gαi/o protein-coupled mechanisms that inhibit neurotransmitter synthesis and release. Activation of H4R reduces forskolin-induced cyclic AMP production, subsequently triggering MAPK pathway activation and increasing intracellular calcium mobilization [42].

On the other hand, endogenous histamine levels from tissue or exogenous histamine levels from food are controlled by two main enzymatic degradation pathways: methylation by histamine-N-methyltransferase (HNMT) and oxidative deamination by DAO. HNMT is a cytosolic protein that degrades intracellular histamine, especially in kidney, liver, spleen, colon, prostate, ovaries, spinal cord, trachea, and respiratory tract. It catalyzes the methylation and inactivation of intracellular histamine. HNMT can be synthesized intracellularly or obtained from the extracellular environment via receptor binding or membrane transporters. In contrast, DAO, a secreted enzyme, is located in tissues such as the small intestine, colon, placenta, and kidney, and is responsible for the elimination of extracellular histamine [49]. Within the intestine, DAO activity progressively increases from the duodenum to the ileum, mainly localized in the intestinal villi. This distribution highlights DAO’s key role in metabolizing dietary histamine at the intestinal epithelium, protecting the body against exogenous histamine from food or intestinal microbiota [12]. It should be noted that the use of the DAO knock-out (KO) mouse to study the role of DAO in the degradation of exogenous histamine is increasingly significant and could provide novel insights [50]. HNMT and DAO have similar affinities for histamine, although HNMT shows a slightly lower affinity. On the other hand, DAO can metabolize other biogenic amines such as putrescine and cadaverine, although it preferentially targets histamine, while HNMT is highly selective for histamine [7]. Therefore, an imbalance between the synthesis, release, signaling, and metabolism of histamine under physiological and pathological conditions will result in HIT.

## 3. Diagnostic Approach

### 3.1. HIT Diagnosis

There is a lack of consensus regarding the approaches used in diagnosing HIT. Despite the plethora of commercial tests, a clinically available, validated biomarker for histamine sensitivity has not yet been developed [8]. The validity of serum DAO activity levels has limitations, such as intra-subject variability [51], and mixed results regarding the relationship with HIT symptoms [8]. In turn, the skin-prick test does not necessarily reflect histamine degradation at the intestinal level and cannot differentiate between histamine intolerance and other allergic conditions [52], while the use of the histamine challenge test is limited because of the risk of adverse events and the absence of a standardized protocol, and stool histamine levels are not considered reliable due to the fact that some intestinal bacteria secrete histamine [8]. Therefore, the diagnosis is based on the clinical assessment and response to a low-histamine diet and reintroduction. Detailed anamnesis is key to understanding the patient’s medical and psychosocial history. Relevant questions include symptom description, duration, and frequency, as well as timing of symptoms to food intake [53]. Specific inquiries about typical problematic foods, such as alcohol, tomatoes, leftovers, fermented foods, cheese, and meats must be considered [53]. In addition, potential associations between symptom onset and medical or lifestyle changes, as well as identification of other factors exacerbating symptoms (e.g., emotional disturbance, menstrual cycle, seasonal allergy, exercise) should also be considered [53]. The symptoms associated with HIT are nonspecific and diverse. Among the gastrointestinal symptoms, bloating has been reported to be the most frequent, followed by postprandial fullness, diarrhea, abdominal pain, and constipation [13]. Among the extraintestinal symptoms the most common are cardiovascular, including dizziness and headache, respiratory, including rhinorrhea, nasal congestion and sneezing, and dermatological, including pruritus and flush [13]. Depending on the clinical picture, the patient should be assessed by specialized physicians to pursue standard medical treatments, such as a clinical immunologist/allergist (for IgE-mediated food allergy mast cell disease), a gastroenterologist (for inflammatory bowel disease, irritable bowel syndrome, and dyspepsia), and a dermatologist (for urticaria, angioedema, and pruritus) [54].

**Figure 3:**
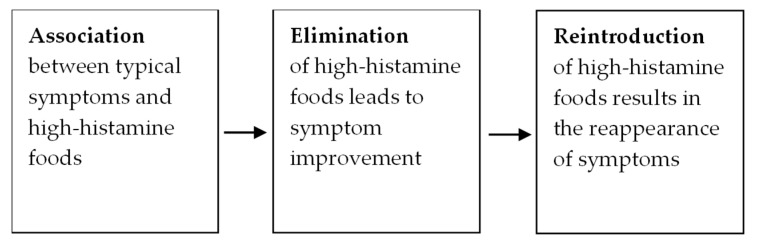
Steps of HIT diagnosis.

The main steps of HIT diagnosis are summarized in Figure 3.

Food and symptom diaries can help identify patterns between dietary intake and symptoms. Additional variables (such as stress, medication, food supplements, physical activity, and menstruation) should be included, based on relevance to each patient. Four weeks is sufficient time to determine if there are any patterns between intake and symptoms. Long-term diaries can exacerbate food and symptom hypervigilance.

### 3.2. Disordered Eating

The patient’s relationship with food is an essential component of the assessment. Elimination diets are contraindicated for patients who are already on a restricted diet, have a history of eating disorders or may be avoiding food due to fear [55]. Fear, food restriction, and worsening symptoms can become a vicious cycle leading to highly restricted diets. In this case, the treatment should focus on normalizing the patient’s relationship with food and diet expansion.

A screening tool for food sensitivity fear in the histamine intolerance population has not been developed. The Nine-Item Avoidant/Restrictive Food Intake Disorder (NIAS) questionnaire has been used to screen for excess dietary restriction in gastrointestinal disease [56]. However, recent research has shown that the NIAS may over-identify and pathologize adaptive restrictions. The Fear of Food Questionnaire has been validated in a patient population comprising adults with inflammatory bowel disease, irritable bowel syndrome, and emetophobia [57].

Unfortunately, this screening tool focuses on gastrointestinal symptoms and does not include pivotal behaviors, such as the time spent engaging in food/health-related research. In the absence of validated screening tools, clinicians can incorporate targeted questions into their assessment related to the patient’s relationship with food. In addition to documenting which foods are avoided, it is crucial to explore patients’ mindsets and reasoning behind the restrictions. Patients may restrict certain foods for judicious reasons, such as sound medical advice, objective observation, and systematic experimentation. However, fear and misinformation are key drivers for many patients.

Elimination diet benefits and risks should be reviewed with patients to help them decide if they would like to proceed. Elimination diets can lead to long-term consequences, including malnutrition, social isolation, disordered eating patterns, and additional stress [58]. Additionally, sustained commitment is needed to follow through with an elimination diet and subsequent reintroduction. Initial motivation often wanes, and abandoning the process may have serious consequences.

## 4. Therapeutic Approaches

### 4.1. Dietary Management

The main management strategy for HIT is a diet low in components that may increase circulating histamine levels [59,60]. Available research looking at the diet’s efficacy is limited due to lack of consensus over cut-off levels, absence of placebo-controlled trials, inconsistent histamine content of foods, and differences in HIT diagnostic criteria [32,54,61,62,63,64,65,66]. Nonetheless, according to the available clinical evidence, adherence to a low-histamine diet has been reported to be effective in reducing gastrointestinal, dermatological, and neurological symptoms [8,21,67].

A suggested low-histamine diet has been designed based on a consensus from the limited research available and further evidence of these specific foods containing components which may increase circulating histamine levels [21,32,61,63,65,68,69,70,71,72]. Several versions of the low-histamine diet circulate on the internet. The discrepancies are largely due to anecdotal evidence. For example, there is a high variability on the histamine content of fermented foods depending on the quality of the raw material, the manufacturing process and storage conditions, and the bacterial strains used [36,73].

HDC, produced by microorganisms, converts histidine (an amino acid) into histamine. Therefore, advice around choosing food processing methods and optimal food hygiene practices designed to prevent microorganism growth are vital in preventing higher levels of histamine accumulating in food. It is important to note that once histamine is in the food it cannot be removed. Table 1 summarizes the existing data about histamine-rich foods, foods that have been reported to interfere with the DAO enzyme, and foods with suggested histamine-releasing capacities. It is important to note that the mechanism responsible for this histamine-releasing capacity has not been elucidated and the existing evidence is inconclusive [34,36].

According to the criteria defined in Section 3.1 a 4-phase method has been proposed to identify HIT based on controlled food intake. Thus, the benefits of dietary restrictions at each phase can be evaluated in order to establish the appropriate tolerance level.

- PHASE 1: A 4-week dietary restriction of foods which may increase histamine levels in the body.
- PHASE 2: 1–2 week reintroduction of small portions of higher histamine-containing foods.
- PHASE 3: (if phase 2 is tolerated) 1–2 week trial of larger portions of histamine containing foods.
- PHASE 4: Continue with tolerated histamine intake and add histamine-releasing and -inhibiting foods.

In addition, there are a large number of drugs that can alter histamine levels, either by promoting the release of histamine contained in mast cells and basophils (e.g., morphine of animal origin, codeine, and acetylsalicylic acid) or by blocking the DAO enzyme (e.g., metamizole, clavulanic acid, and pentamidine) [7,75]. Therefore, clinical intervention will be necessary to monitor concomitant medication in patients with HIT.

After histamine tolerance levels have been established, long-term tolerance should be periodically re-evaluated as factors as such medications, changes to the microbiota, small bowel health, alcohol consumption, and menstruation may change tolerance levels [23,72,76,77]. When patients seek dietary counseling for HIT, they often want precise details on foods to avoid and exclude. Unfortunately, exact lists are not possible due to the lack of scientific research and the inherently inconsistent nature of histamine and other biogenic diamines in food. In medical areas that are poorly defined, such as HIT, the professional’s counseling skills become paramount. For example, professionals can help patients learn to tolerate the ambiguity of not having an exact diet plan. As discussed, continuing unnecessary diet restrictions can have long term negative impacts.

### 4.2. DAO Supplementation

As discussed, decreased DAO enzyme activity levels in the small intestine may allow histamine to be absorbed into circulation. Therefore, oral supplementation with DAO is another treatment approach. Degrading intestinal histamine to safe levels may reduce the symptoms associated with histamine buildup [78].

There are currently numerous food supplements on the market that contain DAO, which can be obtained from different natural sources. Most supplements are formulated from DAO of animal origin (mainly porcine kidney), but there are also products formulated from DAO of plant origin (Lathyrus sativus) and even from recombinant microorganisms (*Yarrowia lipolytica*, which is comparable to human and porcine DAOs) [79,80,81]. Most supplements with DAO only contain the enzyme, but some supplements incorporate vitamins and coenzymes such as vitamin C and catalase [80,82].

DAO oxidatively deaminates several biogenic amines, including histamine. The activity, selectivity and stability of the histamine degradation process plays an important role in the effectiveness of oral DAO supplementation [83]. When histamine and other amines (putrescine, etc.) are degraded by the catalytic activity of DAO, hydrogen peroxide (H_2_O_2_) is produced, which can be toxic to cells. The use of cofactors such as catalase and vitamin C decomposes H_2_O_2_ (hydrogen peroxide) into O_2_ and H_2_O, favoring the activity of DAO enzymes [80]. Thus, oral supplements formulated with DAO from pig kidney and vitamin C have shown a greater capacity to degrade histamine than those formulated only with DAO [79]. In order to determine the ideal conditions for DAO catalytic activity and the possible physiological control mechanisms, it is necessary to study its degradation kinetics, using histamine as the substrate in this case. Most oral supplements on the market present a similar kinetic curve, in which DAO enzymatic activity increases with increasing histamine concentrations, until an inhibition of the substrate is recognized, from which point, the enzymatic activity decreases.

DAO is active in the intestinal mucosa. In vitro studies have shown that DAO is not absorbed from the intestine into the bloodstream [84]. Hence the recommendation is to take supplements with meals, as this is the optimal timeframe to metabolize histamine in the digestive tract, thus minimizing its absorption.

Oral DAO supplementation has been shown to improve histamine intolerance symptoms (Table 2). The DAO supplement tested in the currently available studies was obtained from porcine kidneys, and some formulations also included vitamin C [39] or vitamin C and catalase [85], but there is currently no clinical data on DAO from plant or microbiological origin.

Except in the study by Komericki et al., in which the DAO supplement was administered with histamine-containing tea, the supplement was administered before meals. In some studies, before lunch and dinner [38,86]; in others, before breakfast, lunch, and dinner [13,87,88]; and in the study by O’Connor et al., only before the main daily meal. In addition to the administration pattern of the DAO supplement, huge differences exist between studies in terms of study designs, intervention duration, and the participant’s symptom profile. In most of the studies, patients with general histamine-associated symptoms were evaluated, but in the study of Yacoub et al. [86], the patients included had chronic spontaneous urticaria, while in the study of Izquierdo et al. [87], they had a diagnosis of migraine, and in the study by Okutan et al. [88], fibromyalgia. There are also differences in the criteria to define HIT. In the study by Komericki et al. [39], a histamine provocation test was used to select the study participants, while, in others, the patients were selected according to HIT symptoms after excluding other conditions. In some studies, the serum level of DAO was used as a selection criteria [37,87]. However, there is a certain degree of controversy about the validity of the serum DAO enzyme activity as a diagnostic tool. While some studies have proposed the potential usefulness [38,66], others have obtained inconclusive results [51,89,90].

Regardless of differences in study designs and diagnostic criteria, all the studies have reported significant reductions of HIT-associated symptoms as a result of DAO supplementation. Nevertheless, it is necessary to reach consensus criteria in critical aspects of HIT, such as in diagnosis, to be able to generate further clinical evidence to strengthen the scientific knowledge about HIT and its management.

### 4.3. Pharmacological Interventions

A proper therapeutic approach to HIT requires a differential diagnosis to exclude gastrointestinal diseases or allergies. In the absence of concomitant conditions, the therapy is based on dietary management, but in some cases, adjuvant therapy with H1R and H2R antagonists has been described [7]. Although it has been described that most antihistamines have no effect on DAO activity, there are some exceptions, such as DAO inhibition by cimetidine [91]. Moreover, the additional benefit of antihistamines has not been proven in patients following a low-histamine diet [92]. For this reason, antihistamines are mainly used in acute or severe cases, or when it is not possible to follow a low-histamine diet [8], or when the administration of drugs that may release histamine is unavoidable [93]. Antihistamines may also be used for assessment. A short-term trial of antihistamines has been suggested to evaluate if blocking H1/H2 receptors impacts clinical symptoms [52]. The specific antihistamine should be chosen based on the clinical picture, such as H2 blockers to address nausea/vomiting or H1R blockers for flushing [8,52].

### 4.4. Psychosocial Considerations

When patients seek dietary counselling for HIT, they often want a definitive list of foods to avoid and exclude. Unfortunately, an exact list is impossible due to the lack of scientific research and the inherently inconsistent nature of histamine and other biogenic diamines in food. In medical areas that are poorly defined, such as HIT, the provider’s counselling skills become paramount. For example, patients should be supported to learn new skills to tolerate the ambiguity of systematically experimenting to find their best treatments, rather than definitive direction from health professionals.

Non-food sensitivity reasons for improvement on an elimination diet must be considered. These reasons include, but are not limited to:

- Healthier diet: Patients with a poorly balanced diet (skipping meals, consuming highly processed foods, etc.) may pay more attention and adopt healthier patterns when they start an elimination diet.
- Placebo effect: Expecting benefit from a treatment is a powerful neurobiological influence.
- Treatment effect: Patients often feel better when they receive personalized support from a caring health provider.
- Natural fluctuations: HIT symptoms may wax and wane for unknown reasons. Patients usually make changes (such as an elimination diet) when their symptoms peak, and they may have felt better without intervention.
- Sense of control: Having a treatment plan (such as an elimination diet) can give patients a sense of control over difficult-to-manage symptoms, which can reduce anxiety and improve well-being.

The nocebo effect (expecting something to be harmful) can make food reintroduction difficult. Conditioned food sensitivity (a negative physiologic response prompted by fear/negative expectations rather than by the chemical makeup of the food) is an example of the nocebo effect. When patients are stuck in a trap of fear, food restriction, and worsening symptoms, they may attribute any unpleasant sensations during the reintroduction phase to the new foods. This trap makes diet expansion difficult, and providers should provide thorough follow-up to help patients approach food reintroduction objectively.

## 5. Conclusions

HIT is a food intolerance or non-immunological food reaction in which a variety of clinical symptoms may occur after the ingestion of foods containing or promoting the release of histamine. The purported mechanism is low activity or blockade of DAO in the intestinal lumen. The diagnosis of HIT is hampered by the absence of a validated biomarker and relies on the clinical evaluation, specifically, the response to a low histamine diet and reintroduction of restricted foods. At a practical level, the 4-phase method described above helps identify the histamine intolerance/tolerance level so a personalized dietary plan can be developed. Oral DAO supplementation has been shown to potentially contribute to the degradation of histamine at the intestinal lumen, but existing evidence shows that its histamine-degrading activity could vary depending on the presence of cofactors such as vitamin C, as well as on the origin of the enzyme. Limited clinical evidence suggests a positive role for both personalized dietary management and oral DAO supplementation in the control of symptoms associated with HIT. However, further clinical research is required to determine the potential of these dietary interventions and especially its adaptation to the particular conditions of each patient.

## Figures and Tables

**Table 1:** Foods commonly excluded in HIT diets. Adapted from [7,21,74].

	High Histamine Foods	Histamine Liberator Foods	DAO Inhibitor Foods
Fruits and vegetables	Tomato, spinach, fermented vegetables, eggplant, avocado	Lemons, limes, pineapples, kiwis, and papayas are often culprits	Bananas, citrus fruits, strawberries, pineapple
Proteins	Fermented, cured, smoked or dried meat. Blue fish, canned or preserved fish	Egg white, cow’s milk, seafood	-
Drinks	Alcohol-containing drinks	Coffee, tea, alcohol-containing drinks	Coffee, tea, alcohol-containing drinks, energy drinks
Other	Soy fermented products, yoghurt, vinegars, cheeses (cured, semi-cured or smoked)	Chocolate, cocoa, licorice, peanuts and walnuts	Chocolate

**Table 2:** Clinical studies evaluating DAO supplementation.

Symptom Profile	Design	Intervention Duration (Days)	Sample Size	Main Results Reported	Reference
General HIT symptoms	Randomized double-blind placebo-controlled cross-over study	-	39	Reduction in histamine-associated symptoms compared to placebo	[39]
General HIT symptoms	Retrospective observational study	14	14	13 out of 14 patients reported improvement in at least one of the HIT symptoms	[38]
Chronic Spontaneous Urticaria (CSU)	Randomized double-blind placebo-controlled cross-over study	30	20	Reduction of 7-Day Urticaria Activity Score (UAS-7). Reduction in antihistamine dose	[86]
Episodic migraine	Randomized double-blind placebo-controlled study	30	100	Reduction in migraine episodes duration and triptan intake	[87]
General HIT symptoms	Open-label interventional pilot study	28	28	Reduction in all HIT-related symptoms	[37]
General HIT symptoms	Open-label interventional study	28	82	Reduction in all HIT-related symptoms	[85]
Fibromyalgia	Randomized double-blind placebo-controlled study	56	100	Reduction in fatigue, anxiety, depression, burning, rumination, magnification and helplessness compared to baseline	[88]
